# Oral Perampanel for the Treatment of Super-Refractory Status Epilepticus

**DOI:** 10.1155/2019/8537815

**Published:** 2019-04-09

**Authors:** Abdallah Rahbani, Grace Adwane, Najo Jomaa

**Affiliations:** ^1^Department of Neurology, Lebanese University, Lebanon; ^2^Department of Neurology, Epilepsy and Clinical Neurophysiology Unit, Lebanese Hospital University Hospital, Beirut, Lebanon

## Abstract

A 72-year-old man presented with a super-refractory partial status epilepticus following evacuation of a right subdural hematoma. He failed to respond to treatment with high doses of intravenous valproic acid, Levetiracetam, Lacosamide, Thiopental, and Midazolam. He was given 4 mg of Perampanel orally via nasogastric tube and the dose was rapidly increased to 8 mg after 12 hours with cessation of clinical and electrical seizures. Other antiepileptic drugs were tapered progressively with an excellent clinical outcome.

## 1. Background

This is a case of super-refractory status epilepticus which responded to high doses of Perampanel, therefore highlighting the role of AMPA receptor antagonists in prolonged status epilepticus and the beneficial role of Perampanel in these special cases.

## 2. Clinical Presentation

A 72-year-old hypertensive and diabetic patient presented to the emergency department with left arm numbness and weakness ten days following evacuation of right subdural hematoma. Computed tomography scan of the brain showed partial regression of the right subdural hematoma and its mass effect but with a new hyperdense component suggesting acute bleeding. This was completed by a magnetic resonance imaging of the brain that confirmed the presence of an associated cortical edema. The patient was admitted to the intensive care unit for monitoring and was started on intravenous dexamethasone and oral Levetiracetam. Three days following admission the patient started to have clonic jerks involving the left face and the left upper limb in addition to rapid deterioration of his level of consciousness. He was given loading dose of intravenous valproic acid without clinical improvement. He was rapidly intubated and started on high doses of intravenous Midazolam with persistence of clinical seizures which necessitated the addition of intravenous Thiopental, and the patient was put on EEG monitoring. The dose of Levetiracetam was rapidly increased to 3000 mg and the patient was kept on intravenous maintenance dose of valproic acid. Even on optimal doses of Thiopental that induced burst suppression pattern on the electroencephalogram, the patient was still having recurrent and brief partial seizures that were clinically and electrically evident. Intravenous Lacosamide was added (200 mg twice per day) without any major effect. 24 hours later, in presence of recurrent seizures even on optimal doses of Thiopental, the patient received 4 mg of Perampanel via a nasogastric tube and another 4 mg was given 12 hours later, which induced total resolution of clinical and electrical seizures. The patient was maintained on 8 mg of Perampanel daily. Sedation was progressively decreased and the patient regained progressively a good level of consciousness and was extubated 72 hours later. Valproic acid, Lacosamide, and Levetiracetam doses were progressively decreased and stopped (see Table 1 for details). The patient was discharged home one week later in a relatively very good clinical condition.

## 3. Discussion

Status epilepticus results from an imbalance between persistent cellular excitation mediated by excitatory neurotransmitters “glutamate” and failure of inhibitory synaptic transmission mediated by gamma-amino butyric acid “GABA” [1–3].

As the status epilepticus continues, it becomes more resistant to treatment secondary to the internalization of the postsynaptic GABA receptors to the cytoplasm, externalization of the NMDA and AMPA receptors to the surface, and change in chloride homeostasis in addition to an excess of the extracellular glutamate leading to perpetuation of the status via the AMPA receptors [4, 5].

This explains the resistance to those antiepileptic drugs having a GABAergic effect and a possible better response to those drugs acting on the neuroexcitatory receptors (NMDA and AMPA receptors). The effect of Perampanel, a noncompetitive AMPA receptor antagonist, as an adjunctive treatment in refractory status epilepticus has not been largely studied, with the exception of few published data demonstrating a variable range of efficacy [6–9].

Our case is another example of this molecule's beneficial effect in super-refractory status epilepticus after failure of five appropriately chosen antiepileptic drugs. This can be encouraging for its use in special cases where response to classic drugs is lacking.

## Figures and Tables

**Table 1:** Chronology of the introduction of antiepileptic drugs and their correspondent doses. Those drugs without significant effect on seizures control were progressively stopped.

Time from seizures beginning.	Valproic acid	Midazolam	Thiopental	Levetiracetam	Lacosamide	Perampanel
5 Minutes	40 mg/kg loading dose followed by 500 mg intravenously every 6 hours					

20 Minutes		0.2 mg/kg loading dose titrated to 1.5 mg/kg/hour intravenously		1.5 g every 12 hours per nasogastric tube		

60 Minutes			3 g/24 hours intravenously till burst suppression on EEG.			

3 Hours					200 mg intravenously every 12 hours	

24 Hours						4 mg per nasogastric tube

36 Hours						4 mg per nasogastric tube

48 Hours		Tapering till stopped	Tapering till stopped			8 mg per day

72 Hours and beyond	Tapering till stopped			Tapering till stopped	Tapering till stopped	Maintenance dose of 8 mg per day
